# THE AMBIVALENCE OF BACHELOR OF NURSING LECTURERS TOWARD MALNUTRITION EDUCATION: A QUALITATIVE STUDY

**DOI:** 10.1093/geroni/igad104.2122

**Published:** 2023-12-21

**Authors:** Debbie ten Cate, Iris Van den Boomgaard, Canan Ziylan

**Affiliations:** Utrecht University of Applied Sciences, Utrecht, Utrecht, Netherlands; The Hague University of Applied Sciences, The Hague, Zuid-Holland, Netherlands; Rotterdam University of Applied Sciences, Rotterdam, Zuid-Holland, Netherlands

## Abstract

Malnutrition in older adults occurs multifactorially and should be prevented interdisciplinary. This requires an improvement in the competencies regarding the integral prevention of malnutrition in students of different Bachelor’s programs, such as dietetics, social work and nursing, who are the future professionals. A precondition is that this education is taught by lecturers who have a positive attitude toward malnutrition education. However, it is unknown what this attitude is. We started our exploration within Bachelor of Nursing lecturers. Therefore, the aim of this study was to gain insight into the attitude of these lecturers regarding malnutrition education. A qualitative study, where semi-structured, in-depth interviews were held with nine Bachelor of Nursing lecturers from four different educational institutes. The interviews were transcribed and thematically analyzed. Two main themes emerged from the data: (1) ‘malnutrition is important’, subdivided in three sub-themes: ‘personal experiences’, ‘nursing experiences’ and ‘theoretical knowledge’, and (2) ‘not acting consequently’, subdivided in four sub-themes: ‘unclear responsibilities’, ‘insecurity regarding own abilities’, ‘depending on experts’ and ‘problems with prioritization’. The results showed that lecturers have a positive perception regarding malnutrition education, but that there is a discrepancy between their attitude and actual behavior. This means that this may lead to difficulties in embedding and providing qualitative malnutrition education in the curriculum. It is advised that lecturers follow train-the-trainer courses to develop and enhance their competencies in providing malnutrition education. Also, an efficient integration of and interdisciplinary approach regarding malnutrition education is advised.

